# Magnetic resonance imaging pontine signal abnormality in neurological Wilson disease: A case report

**DOI:** 10.1002/ccr3.7851

**Published:** 2023-08-25

**Authors:** Bikram Prasad Gajurel, Susmin Karki, Asmita Parajuli, Sunil Babu Khanal

**Affiliations:** ^1^ Department of Neurology Institute of Medicine, Tribhuvan University Teaching Hospital Kathmandu Nepal; ^2^ Maharajgunj Medical Campus Institute of Medicine, Tribhuvan University Teaching Hospital Kathmandu Nepal; ^3^ Department of Internal Medicine Institute of Medicine, Tribhuvan University Teaching Hospital Kathmandu Nepal

**Keywords:** hepatolenticular degeneration, pontine hyperintensities, Wilson disease

## Abstract

**Key Clinical Message:**

WD is diagnosed with the help of a brain MRI, which frequently reveals hyperintensities in the lentiform nucleus. But occasionally, high signals can be seen in the pons, thalamus, and midbrain.

**Abstract:**

Wilson disease is a rare inherited disorder due to impaired copper excretion. The brain MRI mainly shows hyperintensities in the lentiform nucleus. We report the case of an 18 years old female diagnosed with neurological Wilson disease, presenting with uncommon brain MRI hyperintensities, predominantly in the pons, thalamus, and midbrain.

## Graphical abstract

Dorsal pons hyperintensity.

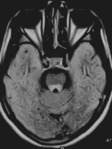

## 1 INTRODUCTION

Wilson disease (WD), also known as hepatolenticular degeneration, is a rare autosomal recessive metabolic disorder caused by a mutation in the ATP7B gene resulting in the accumulation of copper, mainly in the liver, brain, and cornea. It has a prevalence of one in 30,000. It usually occurs among the young population, mainly in the age group of 10–20 years, with male predominance.^1^ Patients may present with various neuropsychiatric symptoms according to the area of the brain involved. At the clinical exam, most patients present with neurological manifestations such as tremors, dysarthria, parkinsonism, dystonia, choreoathetosis, and rarely torsion spasm, in correlation with the affected brain region. Brain MRI helps diagnose WD, revealing most often hyperintensities in the lentiform nucleus. However, in rare cases, the involvement of the midbrain, pons, medulla, and brain lobes showing high signals on brain MRI is possible.^2^ We present a case of Wilson disease showing MRI hyperintensities predominantly in the pons, thalamus, and midbrain.

## 2 CASE REPORT

An 18‐year‐old right‐handed female presented to our hospital with 2 years history of slowly progressive coarse tremors of her limbs which started from her right hand and gradually progressed to whole‐body tremors. She developed progressive disabling dysarthria 8 months prior to the presentation to our hospital. Also, the clinical presentation was completed with progressive difficulty in maintaining her posture and stance while sitting and walking, requiring support for her daily activities (like eating or going to the washroom). She also started to grimace constantly and would always appear happy and smiling, seemingly unfazed by her circumstances. There were no behavioral disturbances. There was no history of headaches, loss of vision, hearing loss, difficulty swallowing, difficulty with comprehension, weakness of limbs, and sensory symptoms in the limbs. Her bowel, bladder, and menstrual history were unremarkable. Her past medical history was not significant. She did not have any other family members with similar illnesses.

**FIGURE 1:**
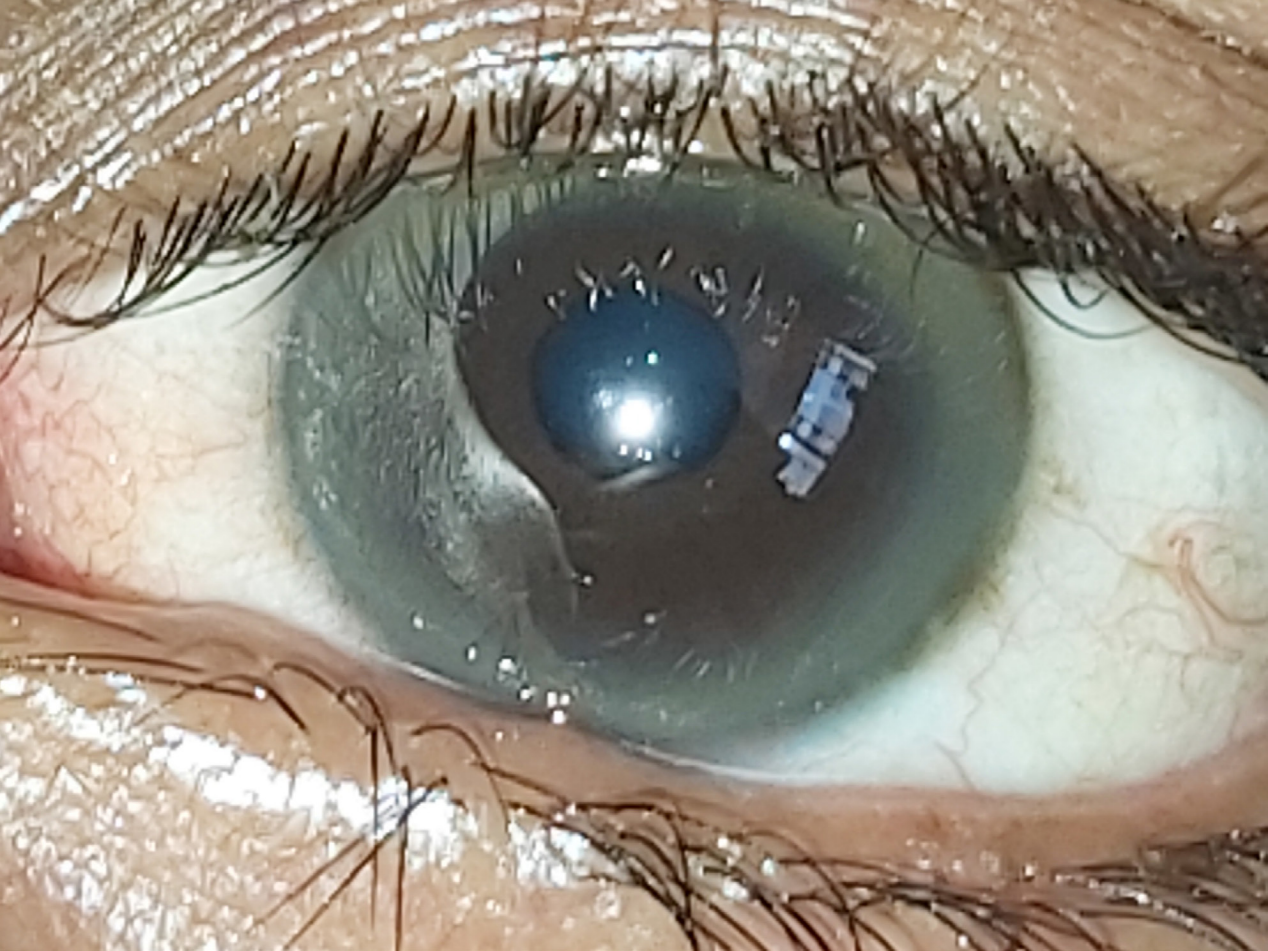
Kayser–Fleischer ring.

On examination, her vital signs were normal. The general physical examination was remarkable only for the Kayser–Fleischer rings in her corneas (Figure 1). The patient was non‐icteric, and organomegaly was absent. Her higher mental function was normal. She had intermittent asymmetrical grimacing movements of the facial muscles. The cranial nerves were otherwise normal. There was coarse action and kinetic tremors in all her limbs which were more prominent on the right side and the right upper limb. There was rigidity across her elbows, wrists, knees, and ankles on both sides, with the right side being more prominently affected. The deep tendon reflexes were exaggerated. The plantar responses were flexor. The power was normal across all joints. There were no abnormal findings on the sensory examination. She could not maintain her stance for over a few seconds due to excessive tremors; thus, gait and cerebellar examinations were impossible.

**FIGURE 2:**
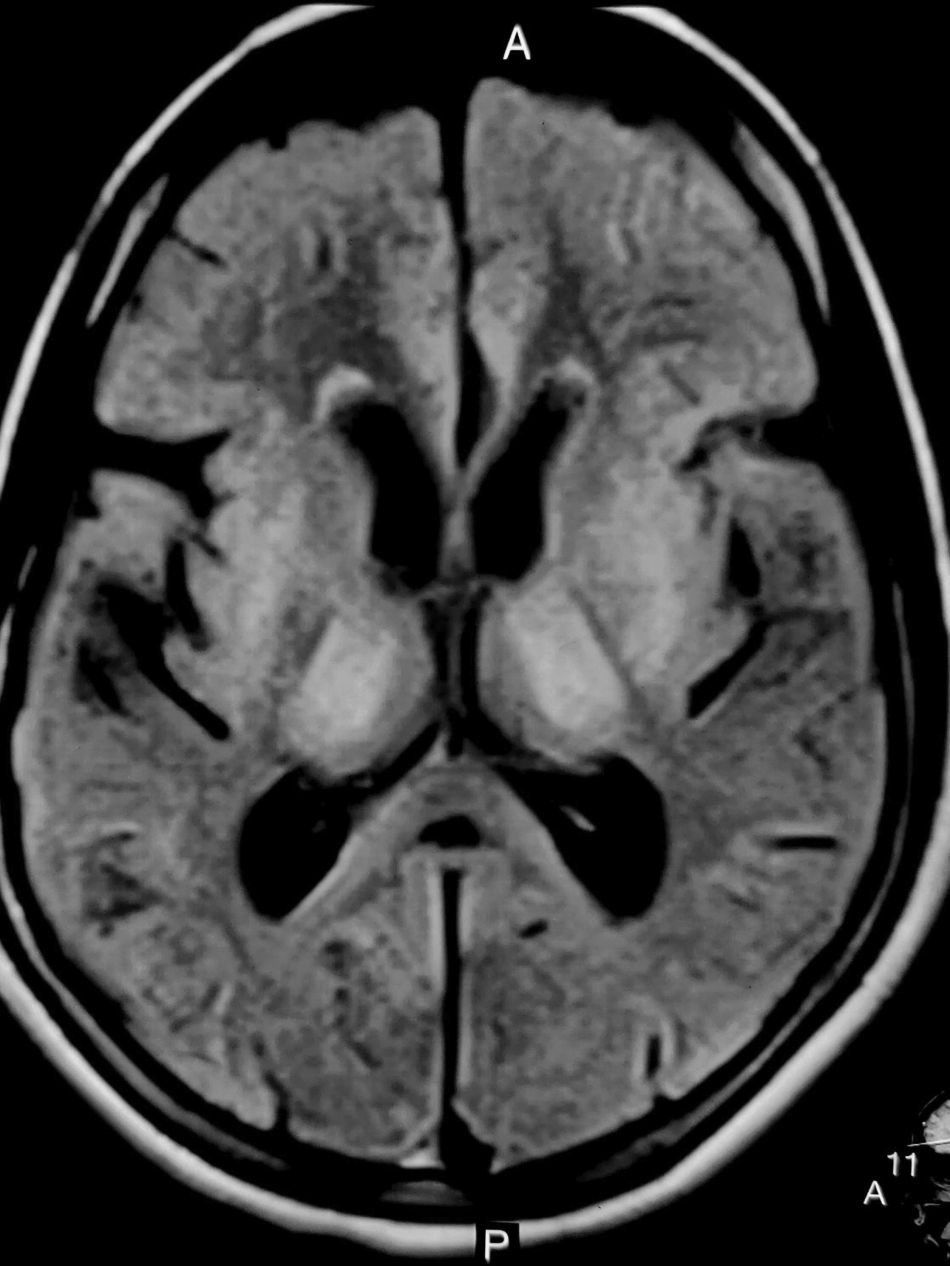
FLAIR hyperintensity in the putamen and thalamus.

**FIGURE 3:**
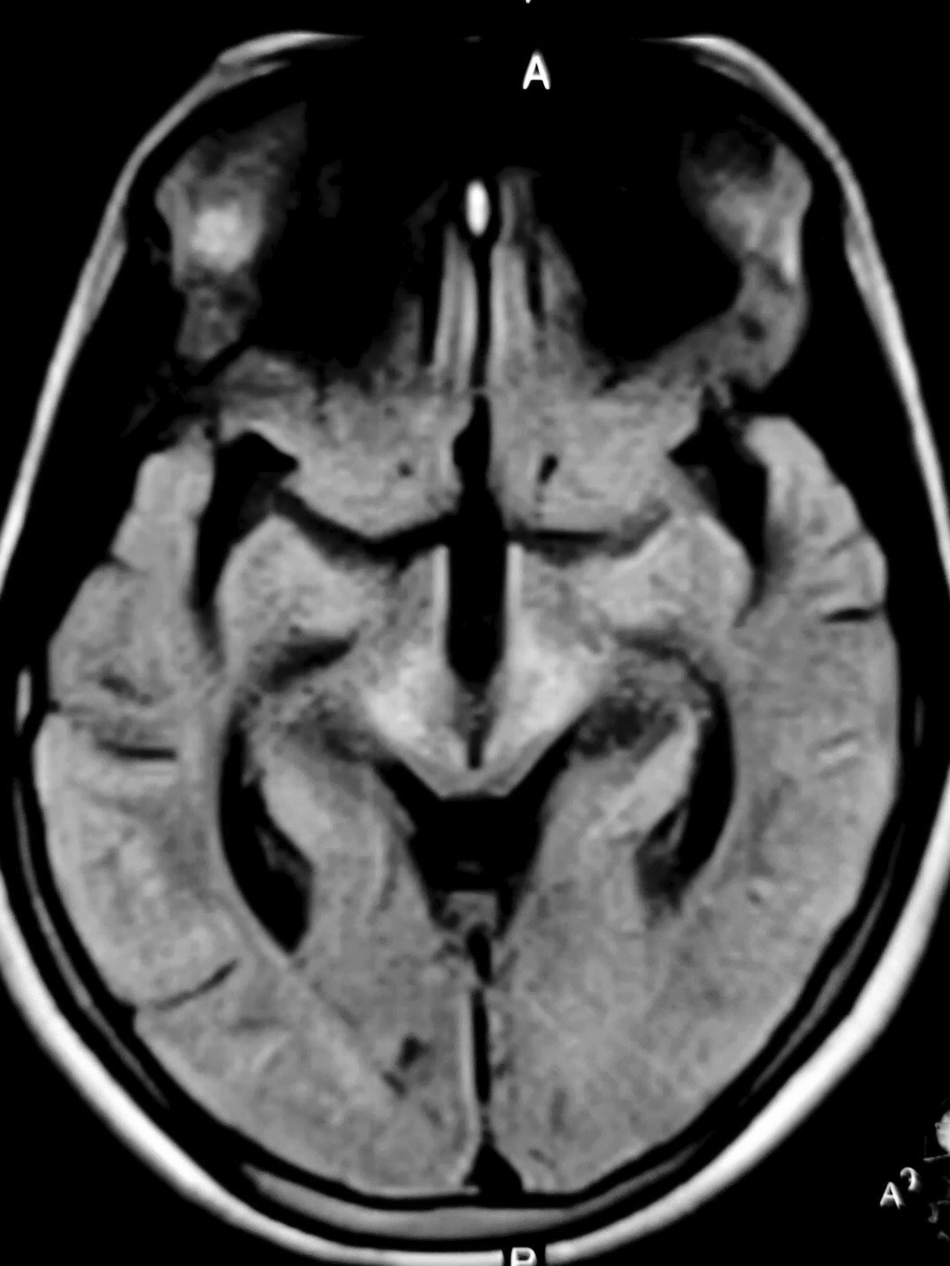
FLAIR hyperintensity in the midbrain.

**FIGURE 4:**
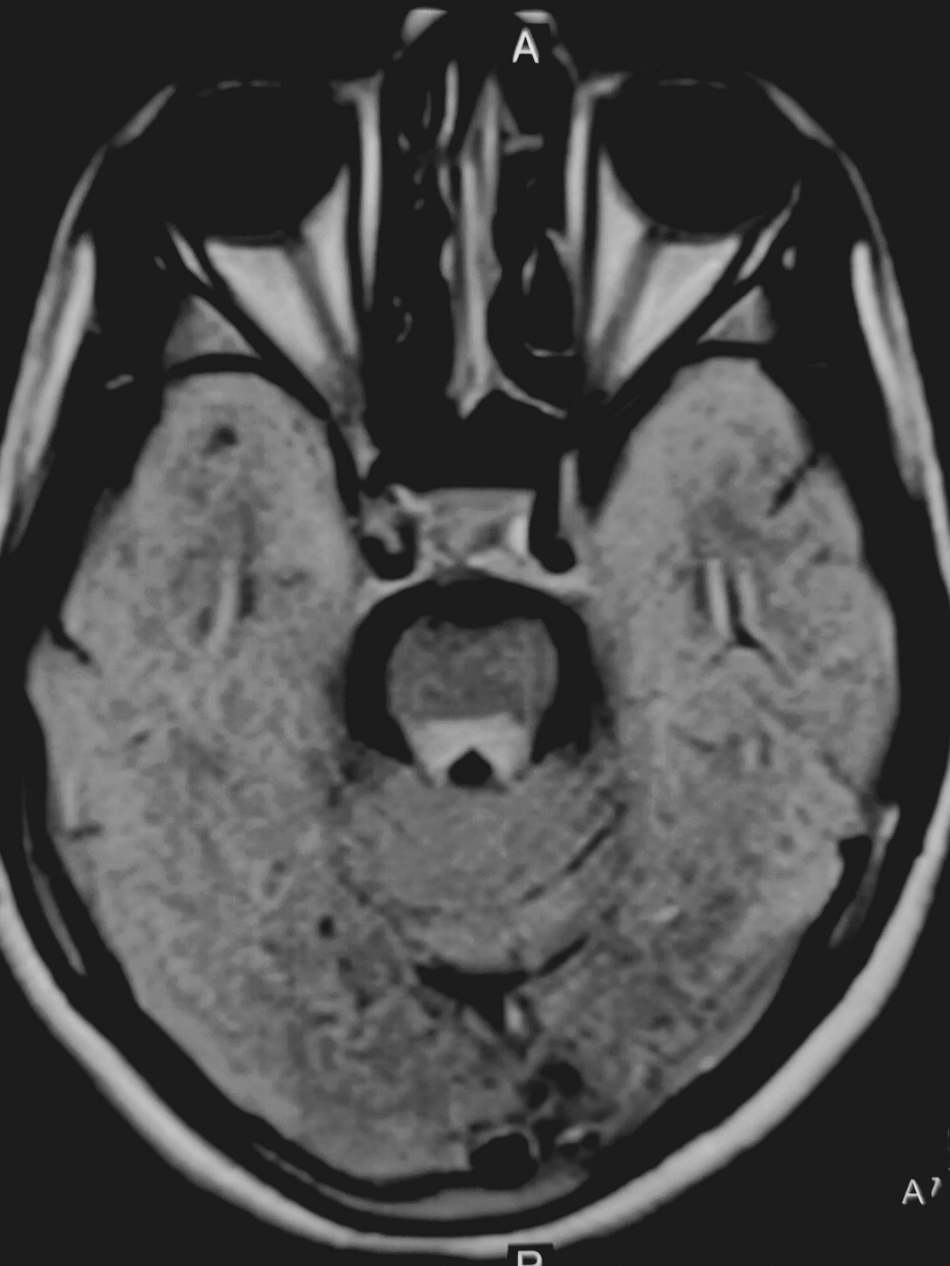
FLAIR hyperintensity in the dorsal pons.

A provisional diagnosis of Wilson disease was made, and investigations were performed. Complete blood count and routine serum chemistry profile that included liver function tests and prothrombin time were normal, ruling out most of the differential diagnosis. The total 24‐h urinary copper excretion was 176.40 micrograms. The serum ceruloplasmin level was 0.10 gm/L. We confirmed the diagnosis of Wilson disease, and magnetic resonance imaging (1.5 tesla) of the brain was performed, which revealed FLAIR high signal intensity in the putamen, thalamus (Figure 2), midbrain (Figure 3), and dorsal pons (Figure 4) with similar features in T2 and normal finding in T1. She was prescribed d‐penicillamine, zinc, and pyridoxine and was advised to follow‐up. However, she was lost to follow‐up.

## 3 DISCUSSION

Wilson disease (WD) is a rare autosomal recessive disorder with a mutation in the ATP7B gene located in chromosome 13, resulting in impaired copper excretion, accumulating copper mainly in the liver, brain, and cornea.^1^ In 2003, the European Association for the Study of the Liver (EASL) put forth the Leipzig criteria for diagnosing Wilson disease (Table 1), which uses clinical and laboratory findings to diagnose WD.^3^ Our patient scored six, and a diagnosis of WD was established.

**TABLE 1:** Leipzig criteria.

Leipzig criteria		
Clinical or laboratory findings		Points
Kayser–Fleischer rings	Present Absent	2 0
Neurologic Symptoms or MRI Findings	Severe Mild Absent	2 1 0
Serum Ceruloplasmin level (g/L)	<0.1 0.1–0.2 Normal (>0.2)	2 1 0
24‐h Urinary copper	>2 × upper limit of normal 1–2 × upper limit of normal Normal Normal, but >5 × upper limit of normal after D‐ penicillamine	2 1 0 2
Coombs‐negative Hemolytic anemia	Present Absent	1 0
Total liver copper level (mmol/g)	>5 × upper limit of normal (>4) Increased (0.8–4) Normal (<0.8) Rhodamine‐positive granules present	2 1 −1 1
Genetic mutation	Present on both chromosomes Present on one chromosome Absent	4 1 0
Total score	Diagnosis established Diagnosis possible; more tests needed Diagnosis unlikely	4 3 2 or less

Taly et al. evaluated 282 patients; 69.1% were found to have neurological symptoms (parkinsonism, dystonia, etc.), 14.9% had hepatic symptoms (jaundice, coagulopathy, etc.), and 3.5% of patients had hepato‐neurological symptoms. Psychiatric symptoms are often the first presenting symptoms, but without neurological or hepatic symptoms, patients get misdiagnosed.^1^ Females present mainly with liver disease, and the mean age of neurological presentation in females is delayed by nearly 2 years more than in males, most probably due to the protective effect of estrogen and iron metabolism difference; in contrast, our patient did not have any hepatic symptoms, clinically as well as biochemically.^4^ The most common presenting neurological symptoms are motor: tremors, incoordination, dystonia, rigidity, and difficulty with fine motor tasks. These symptoms are followed by dysarthria, micrographia, and gait difficulty.^5^ In our case, there were only neuropsychiatric features in the form of tremors, dysarthria, imbalance, rigidity, and emotional incontinence.

Genetic testing for the ATP7B gene is the gold standard for diagnosing WD but is not feasible in our setting. Other investigations include liver function tests, serum ceruloplasmin, 24‐h urinary copper excretion, serum copper, liver biopsy, CT, MRI, etc. The 24‐h copper excretion is increased, while the serum ceruloplasmin, which has high sensitivity and low specificity, is low in WD. Hence, the conditions causing loss or decreased production of ceruloplasmin were ruled out in our patient.^6^ Sometimes a liver biopsy is also considered in the case of hepatic presentation.

In contrast, in the case of neurological presentation, we perform computed tomography (CT) and/or magnetic resonance imaging (MRI) brain to rule in WD and rule out other causes of neurological manifestations. Brain MRI is superior to head CT for detecting gray and white matter abnormalities in Wilson disease, where T1‐ and T2‐weighted imaging is necessary to depict all the neurological abnormalities.^7^ Comparing lesion detection sensitivity in WD, T2 imaging was more sensitive than T1 and FLAIR.^8^ High‐signal intensity T1 changes are mainly seen in cases of hepatic involvement; our patient had no hepatic involvement; hence only had T2 and FLAIR high signals.^9^

Yu et al. did a brain MRI study in 364 patients with neurologic Wilson and found the putamen to be the most frequently damaged brain region regardless of symptoms, followed by the pons, thalamus, caudate nucleus, midbrain, and globus pallidus. Although the pontine hyperintensity in Wilson disease is rare, the study by Yu et al. showed pontine involvement in many cases because they only took neurological Wilson, and more than half of the patients had a diagnosis lag time of more than 1 year; hence likelier to have symmetrical hyperintensities on FLAIR imaging in the pons, midbrain, and cortex.^2^, ^8^ But this might not be true in all cases, as described below from the study by Kim et al. and Mochizuki et al.; Kim et al. evaluated 50 patients with Wilson disease, where few showed hyperintensities in the thalamus and midbrain, whereas only one had pontine hyperintensity.^9^ Similarly, the case series by Mochizuki et al. also did not show any involvement of pons among the three enrolled cases, and involvement of the midbrain was also found only in one case showing the rarity of the pontine hyperintensity in MRI.^10^ A case report by Yousaf et al. also showed similar atypical findings to ours showing atypical MRI features with hyperintense T1 and T2 in the area of the thalamus, midbrain, and pons with minimal involvement of the lentiform nucleus.^11^ The MRI abnormalities characteristic of WD in the midbrain reveals the “face of the giant panda,” a second miniature “panda face” can be seen in the high signal abnormality in the pons. These are rare findings and were also absent in our case.^12^

Multiple medications are approved for treating WD, such as D‐penicillamine, trientine, and zinc salts. Our patient received D‐penicillamine, zinc, and pyridoxine therapy starting at a low dose, with neurological symptoms monitored. A liver transplant is reserved for a patient with acute liver failure or decompensated cirrhosis and is an effective therapy as it restores normal copper excretion and liver function. Dietary copper restriction, symptomatic management of hepatic (esophageal varices, etc.) and neurologic symptoms (parkinsonism, dystonia, focal dystonia, etc.), as well as other multidisciplinary approaches such as speech therapy, physical therapy, and occupational therapy, family screening, and psychiatric consultation, are required for quality of life.

Concerning the reversibility of symptoms, most evidence suggests that both neuropsychiatric and hepatic symptoms improve with appropriate therapy in most patients. However, dystonia may be the least likely of the neurologic symptoms to respond to treatment.^1^ Following copper chelating therapy, the changes in follow‐up MR imaging were strongly correlated with clinical response to treatment showing radiological and clinical improvements.^9^ However, our patient was lost to follow‐up.

## 4 CONCLUSION

WD is a metabolic disorder showing a variable neuropsychiatric, hepatic, or mixed presentation; hence commonly misdiagnosed. Patients suspected of having WD must be screened using a brain MRI, as a timely diagnosis can revert the disease progression. The most frequently involved area of the brain includes the putamen, but, in our case, apart from the putamen, there was predominant involvement of the pons, midbrain, and thalamus, which is rare. A divergence from the conventional brain MRI findings must be recognized since putamen high signals, typically found in WD, may not always be the case.

## AUTHOR CONTRIBUTIONS

**Bikram Prasad Gajurel:** Supervision; writing – original draft; writing – review and editing. **Susmin Karki:** Writing – original draft; writing – review and editing. **Asmita Parajuli:** Writing – original draft; writing – review and editing. **Sunil Babu Khanal:** Writing – review and editing.

## FUNDING INFORMATION

None.

## CONFLICT OF INTEREST STATEMENT

The authors have no conflict of interest to declare.

## ETHICS STATEMENT

Our institution does not require ethical approval for reporting individual cases.

## CONSENT

Written informed consent was obtained from the patient to publish this report in accordance with the journal's patient consent policy.

## GUARANTOR

Susmin Karki is the guarantor of this report.

## Data Availability

Data sharing is not applicable to this article as no new data were created or analyzed in this study.

## References

[1] Mulligan C , Bronstein JM . Wilson disease: an overview and approach to management. Neurol Clin. 2020;38:417‐432. 32279718 10.1016/j.ncl.2020.01.005

[2] Yu XE , Gao S , Yang RM , Han YZ . MR imaging of the brain in neurologic Wilson disease. Am J Neuroradiol. 2019;40(1):178‐183. 30635331 10.3174/ajnr.A5936 PMC7048587

[3] Ferenci P , Caca K , Loudianos G , et al. Diagnosis and Phenotypic Classification of Wilson Disease. Liver Int. 2003;23:139‐142. 12955875 10.1034/j.1600-0676.2003.00824.x

[4] Litwin T , Gromadzka G , Członkowska A . Gender differences in Wilson's disease. J Neurol Sci. 2012;312(1–2):31‐35. 21917273 10.1016/j.jns.2011.08.028

[5] Starosta‐Rubinstein S , Young AB , Kluin K , et al. Clinical Assessment of 31 Patients With Wilson's Disease Correlations With Structural Changes on Magnetic Resonance Imaging. Available from: http://archneur.jamanetwork.com/ 10.1001/archneur.1987.00520160007005 3827691

[6] Taly AB , Meenakshi‐Sundaram S , Sinha S , Swamy HS , Arunodaya GR . Wilson disease: description of 282 patients evaluated over 3 decades. Medicine. 2007;86(2):112‐121. 17435591 10.1097/MD.0b013e318045a00e

[7] Wilson Disease: Findings at MR Imaging and CT of the Brain with Clinical Correlation. 10.1148/radiology.198.2.8596862 8596862

[8] Zhong W , Huang Z , Tang X . A study of brain MRI characteristics and clinical features in 76 cases of Wilson's disease. J Clin Neurosci. 2019;59:167‐174. 30385165 10.1016/j.jocn.2018.10.096

[9] Kim TJ , Kim IO , Kim WS , et al. MR Imaging of the Brain in Wilson Disease of Childhood: Findings Before and After Treatment with Clinical Correlation. Available from: www.ajnr.org PMC8133926 16775300

[10] Mochizuki H , Kamakura K , Masaki T , et al. Atypical MRI features of Wilson's disease: high signal in globus pallidus on T1‐weighted images. Neuroradiology. 1997;39(3):171‐174. 9106287 10.1007/s002340050386

[11] Yousaf M , Kumar M , Ramakrishnaiah R , Vanhemert R , Angtuaco E . Atypical MRI features involving the brain in Wilson's disease. Radiol Case Rep. 2009;4(3):312. 27307828 10.2484/rcr.v4i3.312 PMC4898005

[12] Atalar M , Başpınar N . “Face of the Giant panda” sign in Wilson disease. Turkish Journal of Neurology. 2019;25(3):175‐176.

